# TFP-BD: An image dataset for Traffic Flow and Pedestrian movement analysis on Bangladeshi urban roads

**DOI:** 10.1016/j.dib.2025.111398

**Published:** 2025-02-18

**Authors:** Mohammad Manzurul Islam, Aritra Das, Karib Shams, Md. Rakibul Hasan, Kazi Ferdous Hasan, Mohammad Rifat Ahmmad Rashid, Abdullahi Chowdhury, Md Sawkat Ali, Maheen Islam, Mohammed Shahjalal, Saleh Masum

**Affiliations:** a Department of Computer Science and Engineering, East West University Bangladesh, Jahurul Islam Ave, Dhaka 1212, Bangladesh; b Department of Law, East West University Bangladesh, Jahurul Islam Ave, Dhaka 1212, Bangladesh; c Department of Information and Communication Engineering, University of Rajshahi, Bangladesh

**Keywords:** Pedestrian movement, Traffic anomaly, Traffic flow, Environmental context

## Abstract

In Bangladesh, people sadly are not very much concerned about traffic rules. This study focuses on traffic flow patterns at four locations in Dhaka: Shapla Chattar, Arambag, Bashabo and Abul Hotel. Foot over bridges were used to collect video data, which captured single-lane and double-lane traffic conditions involving different types of vehicles and irregular pedestrian movement. The dataset comprises of 23,678 images extracted from the videos, taken at five different time periods on weekdays. These photos were later annotated using roboflow. This dataset provides a unique view on traffic situations in Dhaka, Bangladesh, by presenting unstructured traffic environments in different conditions at different road junctions. Monitoring vehicle fitness, examining pedestrian behaviour, and measuring vehicle flow in varying environmental contexts (e.g., day, dusk, night, rain) are all possible applications. Researchers can experiment with different machine learning techniques in such traffic environments.

Specifications table

**Table:** 

Subject	Computer Science, Data Science, Machine Learning, Computer Vision and Pattern Recognition.
Specific subject area	This dataset is designed in such a way to develop a comprehensive machine learning model to analyse and predict traffic flow patterns at multiple intersections in Dhaka. This involves collecting and processing large volumes of traffic data, including vehicle counts, pedestrian counts in a varying weather and lighting condition*.*
Type of data	Image (raw and annotated).
Data collection	We captured the videos from four different locations with the same time slots. The time slots are: (i) 7:00 AM to 8:00 AM, (ii) 8:30 AM to 9:30 AM, (iii) 12:00 PM to 1:00 PM, (iv) 6:30 PM to 7:30 PM, and (v) 8:00 PM to 9:00 PM. From the majority of each of these time slots, we collected appx. ten minutes of videos. We have used android mobile phones (Realme XT, Google pixel 5A 5G, and Samsung galaxy S10+) for recording the videos. The videos were captured with 60 fps. After capturing the videos, we extracted one image frame per second. For extracting frames, we have developed and applied an OpenCV python program and obtained 23,678 images. Later all these images were annotated manually using Roboflow. The resolution for both the extracted raw and annotated images is 640×480*.*
Data source location	1. Shapla Chattar (Lat: 23.727075976195344, Long: 90.42173667580597) 2. Arambag (Lat: 23.73099237596215, Long: 90.42120774884675) 3. Bashabo (Lat: 23.740800, Long: 90.426600) 4. Abul Hotel (Lat: 23.754300, Long: 90.415400)
Data accessibility	Repository name: Mendeley Data Data identification number: 10.17632/h8bfgtdp2r.6 Direct URL to data: https://data.mendeley.com/datasets/h8bfgtdp2r/6 The data are in compressed form. Researchers can download the data from the provided link and uncompress them before use.
Related research article	*None.*

## 1 Value of the Data

• This study's dataset offers a valuable collection of images captured from four important roads in Dhaka city. Data were collected from multiple foot over bridges, capturing single-lane and double-lane traffic scenarios. The uniqueness of the dataset is its focus on unstructured traffic environments specific to Bangladeshi roads, showcasing a number of vehicle classes, such as bikes, rickshaws, cars, buses, cycle, mini-truck, truck, CNG and also pedestrian crossing the roads, making it highly relevant for researchers studying traffic patterns in this region.
• To ensure diversity, the dataset was obtained capturing both single lane and double lane views in different time and weather conditions and can help in detecting traffic flow in multiple directions.
• The images were captured in five different time periods (time slots) throughout the day. These time periods reflect the percentage of congestion on the road which can be analysed by different AI models to improve automated traffic systems.
• Environmental contexts (e.g., sunny day, dark night with streetlamps, vehicle headlights, rain) affects how an AI model performs on detecting vehicles and traffic flow. Our dataset encompasses such images having contextual variations that can assist in building a robust AI model.
• Researchers can use this dataset to develop machine learning models for detecting vehicles, identifying pedestrian movements, or analyzing traffic anomalies and apply such models in AI assisted traffic signal and management systems.

## 2 Background

The motivation behind compiling this dataset is to study the complex and unusual traffic flow pattern at different time period of a day in varying weather and lighting conditions in Bangladesh. To our knowledge, no such dataset existed prior to this, making it a pioneering effort in the field. The dataset is unique in its composition, containing both unannotated raw images and annotated images. Researchers can use this dataset to build machine learning models that will be able to detect not only different vehicles and pedestrians but also to recognise traffic patterns in certain environmental contexts, as we collected data in different time periods and weather conditions. The unstructured nature of the traffic on roads of Bangladesh causes a lot of problems, e.g., accidents, traffic congestion, waste of resources and increased pollution. To solve these issues, it is required to study vehicular traffic patterns, unusual movements of pedestrians, unauthorised parking on the road at different times of the day. As our dataset has such images in varying weather conditions (e.g., sunny day, rain, dusk, night), the researcher will be able to test their algorithm for robustness. This dataset will be helpful to introduce an AI controlled traffic system in Bangladesh, leading to ensure a safe and efficient transportation system.

To place our work in the broader context of unstructured traffic environments, we also examine several relevant datasets. For instance, the DATS_2022 dataset [1] consists of mobile-captured images from Indian streets, collected by manually roaming different areas. This dataset includes vehicles, street objects (e.g., traffic signals and road dividers), and even animals, providing a diverse set of scenarios. In contrast, the TFP-BD dataset contains images extracted from fixed-position video footage, focusing on vehicles and pedestrians. Another significant dataset, IDD (Indian Driving Dataset) [2], was collected from rural and urban Indian roads, including single-lane and double-lane environments, similar to TFP-BD. The IDD dataset employs polygon masks for annotation, while TFP-BD uses bounding boxes. These datasets highlight the challenges of traffic analysis in unstructured environments and complement our work, which focuses on the unique traffic conditions of Bangladesh.

## 3 Data Description

**Fig. 1:**
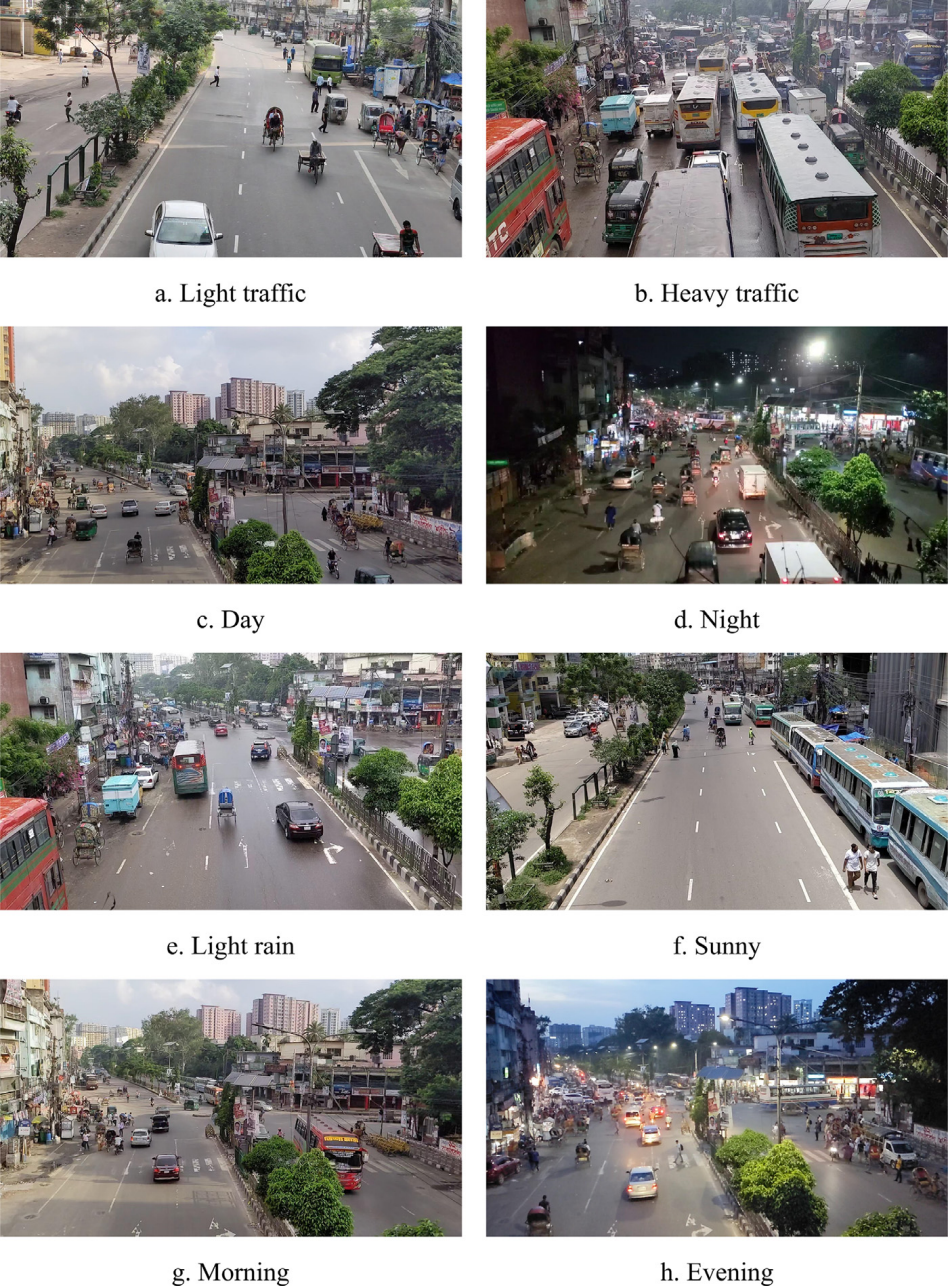
Sample images from the dataset.

The data were collected from four separate locations: (i) Shapla Chattar, (ii) Arambag, (iii) Bashabo and (iv) Abul Hotel. The Dataset consists of 23,678 images extracted from videos of these locations. The videos were 10 minutes long each, with a frame rate of 60 frames per second. The videos were captured from foot over bridges by four different photographers, covering single lane as well as double lane of the road. The videos were captured for 10 minutes each, resulting in 23,678 extracted images. Fig. 1 shows some sample images from the dataset.

Tables 2-9 shows the details of video, images and the weather conditions on which they were captured in different locations. The explanation of the column names of each table are summarised in Table 1.

**Table 1:** Description of columns of Table 2, Table 3, Table 4, Table 5, Table 6, Table 7, Table 8-9.

Column name	Description
Time Slot	Represents the time period of the day while the data were collected.
Traffic Condition	Describes traffic congestions that are labelled as the following 5 categories: (i) light (vehicles move freely with minimal or no delays, maintaining consistent speeds), (ii) light – moderate (slight increase in vehicle density, where movement remains mostly smooth, but occasional slowdowns may occur), (iii) moderate (noticeable increases in vehicle volume, leading to periodic slowdowns and minor congestion), (iv) moderate-high (frequent stop-and-go movements, reduced maneuverability, and longer delays), and (v) high (heavily congested conditions where stop-and-go patterns are persistent, vehicles are closely packed, significant delays are experienced).
Weather	Indicates the weather condition while data was being captured: sunny, cloudy, dusk and night.
Lighting condition	Describes the lighting conditions when the data was collected in the corresponding time slot. The conditions range from daylight to nightlight, rain and transitions such as evening.
No of images	Contains number of images extracted from the captured video.
Length	Represents the length of the video.

**Table 2:** Details of Arambag single lane.

Time Slot	Traffic Condition	Weather	Lighting condition	No of images	length
07:00 - 08:00	Moderate	Clear Sunny	Daylight	609	10:00
08:30 - 09:30	Moderate	Clear Sunny	Daylight	729	12:01
12:00 - 13:00	Moderate-High	Cloudy	Rain	601	10:00
18:30 - 19:30	High	Dusk	Evening	610	10:00
20:00 - 21:00	Light-Moderate	Clear Night	Nightlight	322	5:13

**Table 3:** Details of Arambag double lane.

Time Slot	Traffic Condition	Weather	Lighting condition	No of images	length
07:00 - 08:00	Light	Clear Sunny	Daylight	609	10:00
08:30 - 09:30	Moderate	Clear Sunny	Daylight	568	9:21
12:00 - 13:00	Moderate-High	Cloudy	Rain	610	10:01
18:30 - 19:30	Moderate	Dusk	Evening	634	10:15
20:00 - 21:00	Moderate	Clear Night	Nightlight	301	5:00

**Table 4:** Details of Shapla Chattar single lane.

Time Slot	Traffic Condition	Weather	Lighting condition	No of images	length
07:00 - 08:00	Light	Clear Sunny	Daylight	610	10:00
08:30 - 09:30	Light-Moderate	Clear Sunny	Daylight	610	10:00
12:00 - 13:00	Light	Clear Sunny	Daylight	601	10:00
18:30 - 19:30	High	Dusk	Evening	610	10:00
20:00 - 21:00	Moderate-High	Clear Night	Nightlight	602	10:00

**Table 5:** Shapla Chattar double lane.

Time Slot	Traffic Condition	Weather	Lighting condition	No of images	length
07:00 - 08:00	Light	Clear Sunny	Daylight	607	10:00
08:30 - 09:30	Light	Clear Sunny	Daylight	607	9:58
12:00 - 13:00	Light-Moderate	Clear Sunny	Daylight	612	10:01
18:30 - 19:30	Moderate-High	Dusk	Evening	608	10:00
20:00 - 21:00	High	Clear Night	Nightlight	618	10:00

**Table 6:** Details of Bashabo single lane.

Time Slot	Traffic Condition	Weather	Lighting condition	No of images	length
07:00 - 08:00	Light	Rainy	Daylight	600	10:00
08:30 - 09:30	High	Clear Sunny	Daylight	600	10:00
12:00 - 13:00	Light	Sunny	Daylight	600	10:00
18:30 - 19:30	Light	Dusk	Evening	600	10:00
20:00 - 21:00	High	Clear Night	Nightlight	600	10:00

**Table 7:** Details of Bashabo double lane.

Time Slot	Traffic Condition	Weather	Lighting condition	No of images	length
07:00 - 08:00	Light-Moderate	Rainy	Daylight	600	10:00
08:30 - 09:30	Moderate	Sunny	Daylight	600	10:00
12:00 - 13:00	Moderate-High	Sunny	Daylight	600	10:00
18:30 - 19:30	Light	Dusk	Evening	600	10:00
20:00 - 21:00	Light-Moderate	Clear Night	Nightlight	600	10:00

**Table 8:** Details of Abul hotel single lane.

Time Slot	Traffic Condition	Weather	Lighting condition	No of images	length
07:00 - 08:00	High	Sunny	Daylight	600	10:00
08:30 - 09:30	Light	Sunny	Daylight	600	10:00
12:00 - 13:00	High	Sunny	Daylight	600	10:00
18:30 - 19:30	High	Dusk	Evening	600	10:00
20:00 - 21:00	High	Clear Night	Nightlight	600	10:00

**Table 9:** Details of Abul hotel double lane.

Time Slot	Traffic Condition	Weather	Lighting condition	No of images	length
07:00 - 08:00	High	Rainy	Daylight	600	10:00
08:30 - 09:30	Light	Sunny	Daylight	600	10:00
12:00 - 13:00	Moderate-High	Sunny	Daylight	600	10:00
18:30 - 19:30	Moderate-High	Dusk	Evening	600	10:00
20:00 - 21:00	Moderate-High	Clear Night	Nightlight	600	10:00

**Fig. 2:**
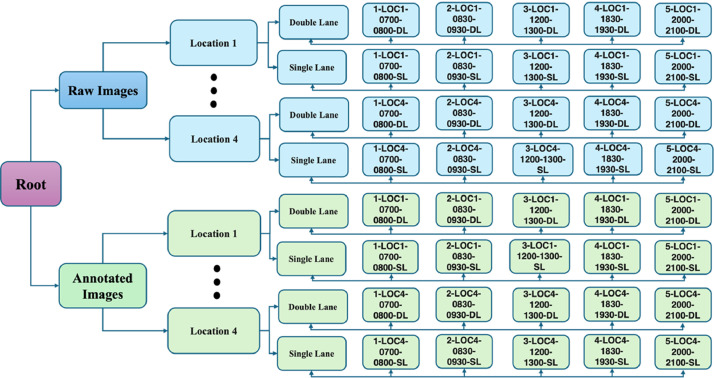
Folder hierarchy.

TFP-BD is an image dataset containing both raw and annotated images extracted from the videos we captured. The root directory in the repository consists of two main folders: “Raw Images” and “Annotated Images”. Both image folders follow the same hierarchy. Each of these folders contain four sub-folders: Location 1 (Arambag), Location 2 (Shapla Chattar), Location 3 (Bashabo) and Location 4 (Abul Hotel). Under each location, there are two sub-folders called “Single Lane”, containing the images for single lane and “Double-Lane”, containing the images for double lane. Finally, each of these sub-folders contain five more sub-folders, named after the time slots based on data capturing timestamp and contain the final images. Fig. 2 shows the folder hierarchy.

Several datasets have been developed to support traffic management and computer vision tasks in the context of Bangladesh and other similar regions with unstructured roads. These datasets vary in terms of size, sources, vehicle classes, and collection methods:

**Poribohon-BD [**3**]:** This dataset contains 9,058 images across 15 vehicle classes. It combines 3,270 images captured using smartphone cameras from roads and highways, 3,997 images sourced from social media platforms (Facebook), and 1,791 augmented images. The combination of online images and augmented data may introduce biases related to data quality and real-world representation.

**Dhaka-AI [**4**]:** Comprising 3,953 images divided into training and testing splits, this dataset spans 21 vehicle classes. The images were primarily extracted from YouTube videos. However, the dataset lacks sufficient information about the diversity of traffic conditions and may suffer from limited contextual variations.

**Vehicle-BD [**5**]:** This dataset consists of 1,085 original images spread across 9 classes. An additional 12,413 images were generated through augmentation, resulting in 13,498 total images. Despite the augmentation, the limited number of original images constrains its generalization potential.

**ANNA [**6**]:** Comprising 1,800 manually selected images, this dataset was built by extracting frames from videos recorded in low-traffic areas across Dhaka (Mirpur and Bashundhara) and Jhenaidah (Khulna division). While it includes multiple locations, the dataset largely represents low-traffic scenarios and lacks contextual diversity.

In comparison, our dataset offers several distinct advantages that make it a more comprehensive and versatile resource for traffic research. With 23,678 manually captured images, our dataset consists entirely of authentic, real-world traffic data without reliance on online sources or augmented data, ensuring unbiased representation. Data was collected under varied weather conditions, traffic congestion levels, and different times of the day, making it adaptable to a wider range of machine learning applications. All images in our dataset have been meticulously annotated by hand, ensuring high-quality labels, which is critical for building reliable machine learning models. These strengths position our dataset as a high-quality resource that addresses key limitations present in existing datasets, supporting more robust and generalizable AI models for unstructured traffic scenarios.

## 4 Experimental Design, Materials and Methods

We collected the data from some busy roads in Dhaka. Though there are a lot of cars (e.g., private cars, bus, rickshaws) on the roads, people are still crossing the road without any zebra crossing. We captured videos of both single and double lanes of different roads from four different junctions in Dhaka city. Then using OpenCV and Python, we extracted images from these videos. The data have been collected in such a way that a researcher can not only use the dataset to decode pedestrian crossing patterns, but also to find the vehicle flow rate, vehicle pattern and so on.

In recent times the machine learning (ML) and AI sector is booming. More people are getting attracted to the fascinating world of ML and AI applications in all areas of daily life. Recommendation and prediction are two widely used application of ML. In order to use ML to predict something accurately and efficiently, we need a good dataset, because the correlation between the dataset quality and machine learning model performance is quite high [7,8].

We can use different ML techniques to detect pedestrians or to find the traffic flow. Although there exists a few traffic related dataset, but to the best of our knowledge, no existing datasets are available that was created considering contextual variations (e.g., weather condition, lighting), flow of traffic in single or double lane throughout the day in several predefined time frames, and unusual pedestrian movements in an unstructured road traffic condition.

### 4.1 Methodology

The dataset plays a very important role in the machine learning models. Practitioners believe the quality and quantity of data is more important than the mathematical model itself, because it has a bigger impact on the accuracy of the ML model applications. This idea is widely known as data centric approach [9]. Generally, the following steps are adopted to create a dataset [10]:

• Selecting samples from real world
• Cleaning data
• Augmenting data
• Labelling data.

**Fig. 3:**
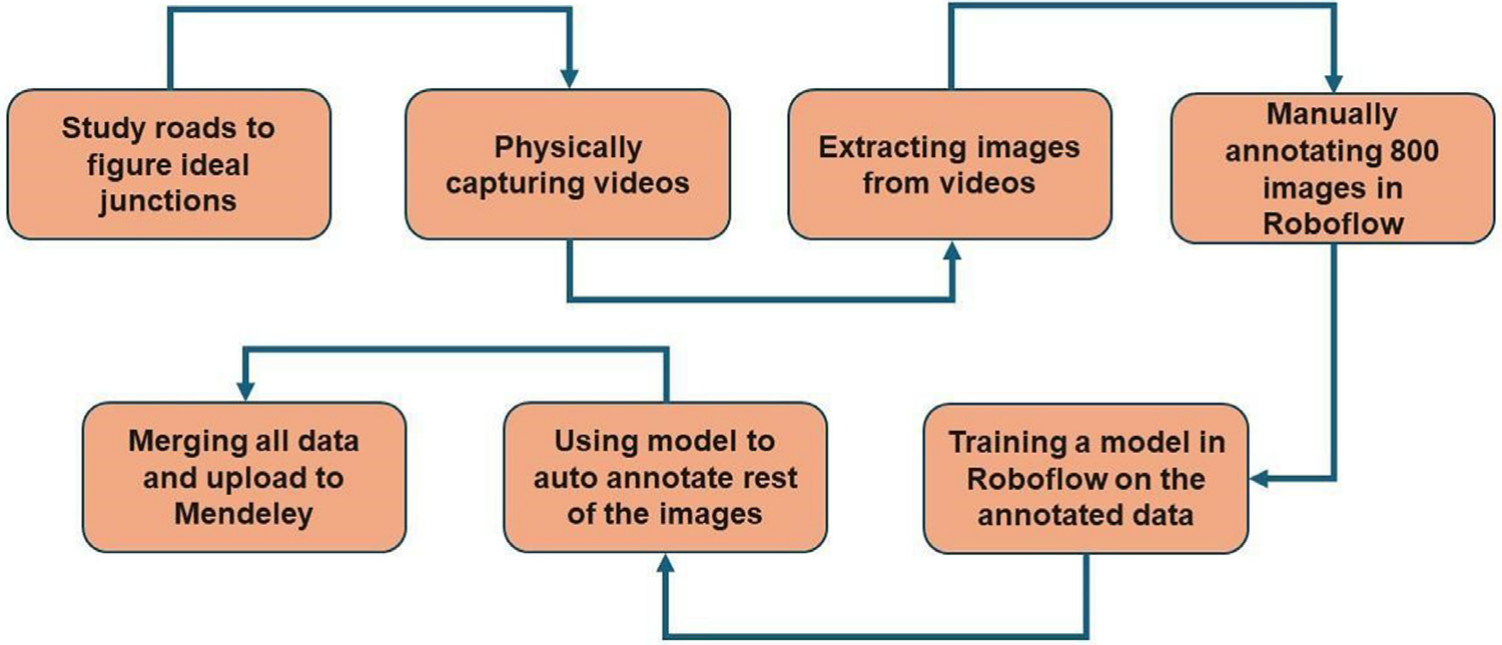
Data collection and preparation steps.

To build our dataset we followed the following steps: at first, we studied different roads to find the ideal spots. Then we physically captured the videos of those roads. After that we extracted frame-by-frame images from the videos. Then we manually annotated all the extracted images using Roboflow. Fig. 3 shows the flow chart of our work process.

### 4.2 Data collection

In the data collection process, we conducted an extensive survey of Dhaka city roads to identify suitable roads for our study. We specifically selected roads equipped with foot overbridges, enabling us to capture high-quality video footage from an elevated perspective, thus ensuring uniformity, safety, and clarity. A crucial criterion for road selection was high traffic density; hence, we included several busy intersections in our study. Subsequently, we designated five different times for video recording to incorporate variability in our data and to examine changes in traffic volume throughout the day, particularly during peak and off-peak hours. We then proceeded to record videos, focusing on both single and double-lane roads. These recordings were made simultaneously at various junctions to maintain consistency across data points. To minimize disruptions in video quality, we ensured that the camera was stable during recording. We employed some smartphone cameras, adjusted to optimal settings to capture the best possible footage. Detailed specifications of the camera and video settings have been previously provided in specification table.

### 4.3 Data preprocessing

**Fig. 4:**
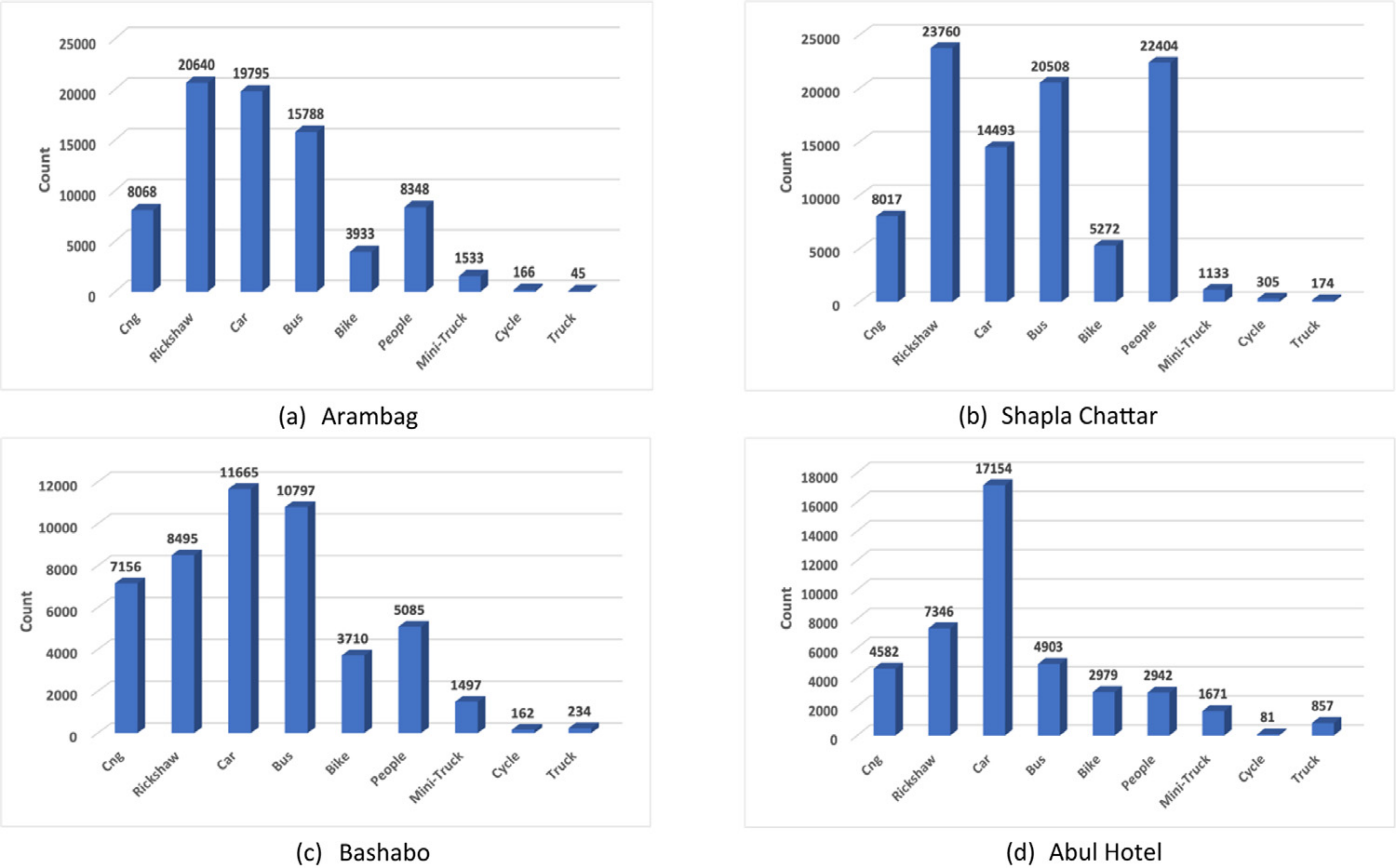
Annotated class distribution at different locations.

**Fig. 5:**
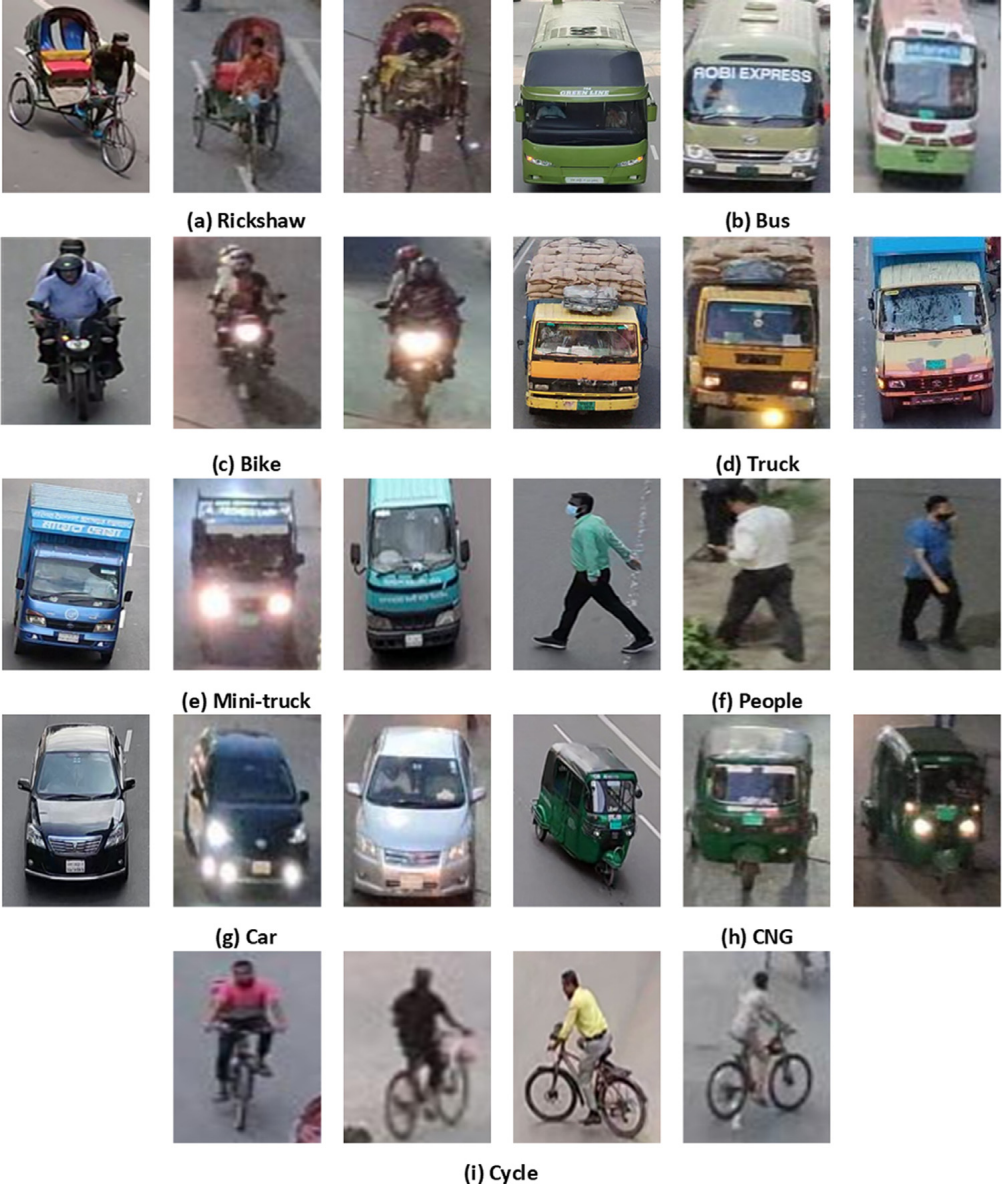
Sample images for different annotated classes.

In the preprocessing phase, frames are extracted from captured videos using OpenCV, with a specific criterion of extracting one image per second from each video. The extracted images were then uploaded to Roboflow [11], a platform used for image annotation, where they were categorized into eight primary folders. These folders were designated as “Shapla Chattar single lane”, “Shapla Chattar double lane”, “Arambag single lane”, “Arambag double lane”,“Bashabo single lane”, “Bashabo double lane”, “Abul Hotel single lane” and “Abul Hotel double lane”, each containing five subfolders representing different time intervals throughout the day. The initial phase of manual annotation involved the division of labour among four team members, each responsible for manually annotating 5920 images carefully. This process resulted in the manual annotation of 23,678 images using Roboflow. Fig. 4 illustrates the annotated class distribution at different locations while Fig. 5 shows the annotated classes.

## Limitations

Constructing a traffic image dataset using only mobile devices presents some difficulties. For instance, mobile phones, while convenient, generally do not match the performance of professional cameras. They tend to produce images with lower resolution, more noise, and reduced clarity. Moreover, capturing images in low light conditions or adverse weather can be particularly challenging with mobile devices, which are often limited in their lighting capabilities. Although resent mobile cameras have excellent built-in image stabilization features, without the aid of a tripod, the video may become shaky or blurry, which can further degrade the quality of the dataset. High-quality video recordings also demand significant storage space, often 2 gigabytes or more per video, necessitating frequent transfers of data to additional storage devices to manage space efficiently. In our dataset, we were careful regarding these limitations and tried our best to create an excellent dataset using high quality camera based android mobile phones (Realme XT, Google pixel 5A 5G, and Samsung galaxy S10+).

In future, we plan to expand the dataset by collecting data from diverse regions across Bangladesh, including urban and rural areas, and from neighboring South Asian countries with similar traffic patterns. Additionally, we will capture seasonal variations and different traffic conditions using weather-resistant systems and edge computing solutions, supported by local partnerships and research grants.

## Ethics Statement

The images for this dataset were collected from publicly accessible spaces, specifically urban roads, where there is no reasonable expectation of privacy. According to ethical guidelines for visual research, capturing images in public spaces typically does not require formal ethical approval, provided that the images are used in a way that does not infringe on individuals' privacy or expose them to harm. The International Visual Sociology Association (IVSA) guidelines support the collection of such data in public spaces without formal consent [12], as long as it is ethically handled and individuals are not specifically identified or harmed through the research process.

Additionally, no approval from local authorities was required as the images were captured in general public spaces where there are no restrictions on photography or videography of public scenes. The data collection adhered to local regulations, and no sensitive or private information was recorded [13]. This work fulfils publishing ethics guidelines.

## Credit Author Statement

**Mohammad Manzurul Islam:** Conceptualization, Supervision, Writing (review and editing). **Aritra Das:** Methodology, Software, Formal analysis, Data curation, Validation. **Karib Shams:** Data curation, Validation. **Rakibul Hasan:** Data curation, Investigation, Writing (original draft), Resources. **Kazi Ferdous Hasan**: Project administration, Data curation. **Mohammad Rifat Ahmmad Rashid:** Supervision, Writing (review and editing). **Abdullahi Chowdhury:** Writing (review and editing). **Md Sawkat Ali:** Validation. **Maheen Islam:** Project administration, Resources. **Mohammed Shahjalal:** Supervision. **Saleh Masum:** Writing (review and editing).

## Data Availability

Mendeley DataBangladeshi Traffic Flow Dataset (Original data).
